# Response to the Letter to the Editor regarding, “Research Note: Sex difference in changes in heterophil to lymphocyte ratios in response to acute exposure of both corticosterone and cortisol in the Pekin duck”

**DOI:** 10.1016/j.psj.2022.102059

**Published:** 2022-07-21

**Authors:** Gregory S. Fraley

**Affiliations:** Terry and Sandra Tucker Endowed Chair of Poultry Science, Purdue University Animal Sciences, West Lafayette, IN 47907, USA

Dear Dr. Taylor,

Thank you for bringing to my attention the Letter to the Editor written by Dr. Paul Cotter concerning my lab's paper, “Research Note: Sex difference in changes in heterophil to lymphocyte ratios in response to acute exposure of both corticosterone and cortisol in the Pekin duck” Poultry Science 101:101914. I appreciate that Dr. Cotter has brought to light some improvements that should have been included in the manuscript. For example, Dr. Cotter raised concerns about the process used to determine the heterophil to lymphocyte ratio. To comply with the Research Note format, and to avoid self-plagiarism from several other manuscripts, we did not adequately describe this methodology. Blood smears were read manually by veterinary pathologists in a double-blind manner at the independent lab, Antech Diagnostics, Inc. (Chicago, IL). The results were verified by a Board Certified avian pathologist. The Avian/Exotic CBC differential from Antech includes: heterophils, bands, lymphocytes, monocytes, eosinophils, and basophils. The heterophil/lymphocyte ratio was calculated as originally described (Gross and Siegel, 1983. Evaluation of the heterophil/lymphocyte ratio as a measure of stress in chickens. Avian Dis. 27:972-979) through dividing the number of heterophils by the number of lymphocytes. Following this procedure, bands (all 0 scores) were not included in the heterophil/lymphocyte ratio. Further, the avian pathologist made no comment about observed abnormal heterophils. It has not been this investigator's observation that including tables of the differentials is common practice for Poultry Science, or any other journal, when presenting HLR values. However, we will consider doing so in future publications. As described in the Methods, the birds were 40 weeks of age when treated, thus it is not clear what age adjustment is necessary.

Dr. Cotter also cited concerns about the baseline value and some peak values for our heterophil/lymphocyte ratios. I agree that baseline ratios should be around 0.5 to 1.0, however, no normal range values have been determined for any CBC component of the modern Pekin duck . The closest relative is the Mallard duck whose normal values are described in the current edition of “Exotic Animal Hematology and Cytology,” by Terry Blackwell. Although the Pekin duck is not a Mallard, all other CBC components were within the normal range for Mallards. Regardless, we admit that the baseline HLR values were slightly elevated. The simplest, and most likely, explanation is that the birds were responding to capture, handling, and blood collection. The suggestion that elevated values are due to infection at the injection site is unreasonable as that could not manifest itself in less than 3 h. Further, the rare pathologies additionally suggested by Dr. Cotter would be unlikely to occur in 40 birds simultaneously. To then show variable HLR changes over a 3-h period, in a treatment-specific manner, further amplifies the unlikeliness of those possibilities. The fact that these were healthy birds is supported by absence of external or internal signs of infection or other pathology in necropsies independent of this experiment which were performed a week later.

This publication described our study that involved the exogenous and pharmacological manipulation of the immune system. As such, the fact that some individual birds responded in a hyperphysiological fashion is not surprising; this is simply the nature of endocrine studies, but we agree that these results are extraordinary. Cellular reserves exist for a time of stress when a very rapid immune response is necessary. The question about the evolutionary adaptations that may make this response possible is an interesting one. However, any discussion would be purely speculative and has no purpose in this forum.

This paper provides further evidence that cortisol has physiological actions which do not simply mirror those of corticosterone. Our laboratory, along with others around the world, have recently been demonstrating that cortisol is likely an important endocrine agent in the bird. This current Research Note is just one small piece of that puzzle. Again, I thank Dr. Cotter for his comments and hope this response has helped to clarify inadequacies in our publication.

Sincerely,

Gregory S. Fraley, Ph.D.

